# A multi‐data ensemble approach for predicting woodland type distribution: Oak woodland in Britain

**DOI:** 10.1002/ece3.7752

**Published:** 2021-06-21

**Authors:** Duncan Ray, Maurizio Marchi, Andrew Rattey, Alice Broome

**Affiliations:** ^1^ Centre for Ecosystems Society and Biosecurity Forest Research Roslin UK; ^2^ Institute of Biosciences and BioResources (IBBR) CNR, Florence Division Sesto Fiorentino (Firenze) Italy

**Keywords:** biomod2, national forest inventory, *Quercus robur*, *Quercus petraea*, species distribution model

## Abstract

Interactions between soil, topography, and climatic site factors can exacerbate and/or alleviate the vulnerability of oak woodland to climate change. Reducing climate‐related impacts on oak woodland habitats and ecosystems through adaptation management requires knowledge of different site interactions in relation to species tolerance. In Britain, the required thematic detail of woodland type is unavailable from digital maps. A species distribution model (SDM) ensemble, using biomod2 algorithms, was used to predict oak woodland. The model was cross‐validated (50%:50% ‐ training:testing) 30 times, with each of 15 random sets of absence data, matching the size of presence data, to maximize environmental variation while maintaining data prevalence. Four biomod2 algorithms provided stable and consistent TSS‐weighted ensemble mean results predicting oak woodland as a probability raster. Biophysical data from the Ecological Site Classification (forest site classification) for Britain were used to characterize oak woodland sites. Several forest datasets were used, each with merits and weaknesses: public forest estate subcompartment database map (PFE map) for oak‐stand locations as a training dataset; the national forest inventory (NFI) “published regional reports” of oak woodland area; and an “NFI map” of indicative forest type broad habitat. Broadleaved woodland polygons of the NFI map were filled with the biomod2 oak woodland probability raster. Ranked pixels were selected up to the published NFI regional area estimate of oak woodland and matched to the elevation distribution of oak woodland stands, from “NFI survey” sample squares. Validation using separate oak woodland data showed that the elevation filter significantly improved the accuracy of predictions from 55% (*p* = .53) to 83% coincidence success rate (*p* < .0001). The biomod2 ensemble, with masking and filtering, produced a predicted oak woodland map, from which site characteristics will be used in climate change interaction studies, supporting adaptation management recommendations for forest policy and practice.

## Graphical abstract

The manuscript provides a method to predict a thematic woodland type within broad habitat woodland classes, using oak dominated woodland as a case study. The approach uses different woodland spatial and non‐spatial data with the biomod2 ensemble model. Good results were obtained by the approach, and the resulting oak woodland distribution map will be used to evaluate the interactions between climate, topography and soil conditions under climate change. This will help develop timely adaptation management plans to reduce impacts.

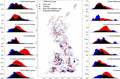

## 1 INTRODUCTION

New evidence in a review (Duffy et al., 2019) of climate change threats on ecosystems reported severe and pervasive impacts on various managed and natural systems. These include food production and agriculture, forestry, ecosystems, and wildlife and involve direct, indirect, and interacting impacts (Seidl et al., 2017). The vulnerability of terrestrial ecosystem components to abiotic stress is in general related to habitat connectivity (Eigenbrod et al., 2015), and for plants, site‐type acclimation and plasticity are also important (Dorado‐Liñán et al., 2019). At a more detailed scale, variation in topography, lithology, and elevation may interact to exacerbate, or alleviate, species vulnerability (Crossman et al., 2012; Pacifici et al., 2015; Synes et al., 2020). This implies the need for explicit knowledge of the spatial distribution of habitat to effectively model likely climate impacts and consider local site mitigation choices for managed ecosystems (Lõhmus et al., 2020; Oliver et al., 2012). However, knowledge on the precise location of specific habitats is often unavailable, difficult to obtain, or of inappropriate resolution (Jongman et al., 2019). Thus, determining where habitat occurs without precise information is a major challenge for management and planning.

In Britain, tree species information for woodland habitats is largely unavailable. Stand‐level forest classification systems (Kusbach et al., 2017; Pojar et al., 1987; Pyatt et al., 2001; Ray et al., 2009) operate at a fine (local) scale, but are commonly limited in spatial extent. The coarse resolution of national (Chirici et al., 2012), regional (Schelhaas et al., 2018), and European scale forest biomes—for example, resources such as EUFORGEN (Ducousso & Bordacs, 2004) the European Forest Genetic Resources Programme; and EFISCEN (Schelhaas et al., 2006) the European Forest Information Scenario Model at a NUTS‐2 level (European regional Nomenclature of Territorial Units for Statistics)—are unsuitable for landscape spatial analysis of managed forest types at subregional levels (Tröltzsch et al., 2009).

Forest may be publicly or privately owned and data availability varies between the two. In privately owned forest, the spatial pattern of dominant tree species within “broad habitat types” is poorly understood, with knowledge being limited to the British (GB) National Forest Inventory (NFI) Indicative Forest Type maps (NFI maps). However, these data are resolved to conifer, broadleaved or mixed woodlands, with no information about particular species. The NFI survey holds a restricted‐access database of woodland sample plots representing 0.6% of Britain's woodland cover from 15,100 1ha plots (Ditchburn et al., 2020). This is a small sample compared with publicly owned forest, where the entire forest estate (32% of woodland area) is inventoried and resulting information is commonly open and accessible for research (https://data.gov.uk ‐ National Forest Estate Sub‐Compartments). Field mapping is expensive to conduct over large areas. High‐resolution hyperspectral image classification is a promising method for reliable identification of individual tree species within woodlands (Hycza et al., 2018), but the narrow spectral bands with high correlation require a large amount of processing, and software is currently expensive.

Species Distribution Models (SDMs) have been successfully used to assess the fundamental niche of a variety of taxa including tree species (Booth, 2018; Di Pasquale et al., 2020; Elith & Leathwick, 2009; Marchi & Ducci, 2018; Pecchi et al., 2019; Thuiller, 2003). In particular, the package “biomod2” (Thuiller et al., 2016) in the R statistical programming language (R Development Core Team, 2020) offers ten algorithms including statistical, machine learning, and classification methods. SDM techniques rely on species occurrence in natural ecosystem distributions, with the assumption that a species/site‐type (climate, soil, topography, competition) dynamic equilibrium occurs. Managed ecosystems are subject to anthropogenic manipulation, and in this context, SDM assumptions of niche theory could be compromised. In Britain, as in many countries, most forests have some history of management despite being designated primary (ancient) or secondary (planted) forests (Bradshaw et al., 2015). The ancient forests of Britain have a complex “cultural landscape” heritage. Oak was a component of mixed natural woodlands before human intervention and clearance. The management of ancient woodlands over many centuries has selected for oak to form a more dominant canopy component than occurred naturally (Rackham, 1989). Over recent centuries, secondary oak woodlands have been planted and managed for timber, and perhaps sometimes on sites that may never have been ecologically suitable for oak woodland.

In this study, we wish to test for the first time whether SDMs trained using public forest inventory data, combined with NFI information, can provide a rapid and tractable method of creating a realistic probabilistic map of managed secondary (planted), and managed primary (termed ancient semi‐natural—with both natural and planted regeneration on ancient woodland sites), oak stands in Britain. Ideally, we would have used the NFI survey data to build the models as they are taken from a random‐stratified sample of woodlands, but data access issues prohibited this. The approach we have used instead uses fine‐scale data from oak woodlands on the public forest estate to predict privately owned oak woodland. We refine the model prediction using regional‐scale information from the NFI sample square reports and the elevation distribution from the NFI sample squares. Knowledge of the distribution of oak woodland types will help us understand site‐type–oak woodland interactions across NFI regions. Approximately 90% of native oak woodland is privately owned (Ditchburn & Ross, 2018), mostly with no information about location. This is important as we need location information to study the effects of climate change on oak woodland sites. This is to develop recommendations for a policy‐led approach, to target adaptation management of oak woodland stands at a regional level. Oak is threatened by a complex decline (Denman et al., 2014; Thomas et al., 2002) associated with site stress (Gibbs & Greig, 1997; Oosterbaan & Nabuurs, 1991) that has intensified with climate change (Rozas & García‐González, 2012). Our study species are *Quercus petraea* and *Q. robur*. These native oaks are selected as they are important broadleaved production species in Britain, with 70 million m^3^ of standing trees forming 28.5% of the standing broadleaved resource (Forest Research, 2020). In addition, oak has an extremely high biodiversity interest, attracting up to 2,300 associated species, over 300 of which are oak obligate species (Mitchell et al., 2019).

## 2 MATERIALS AND METHODS

**FIGURE 1:**
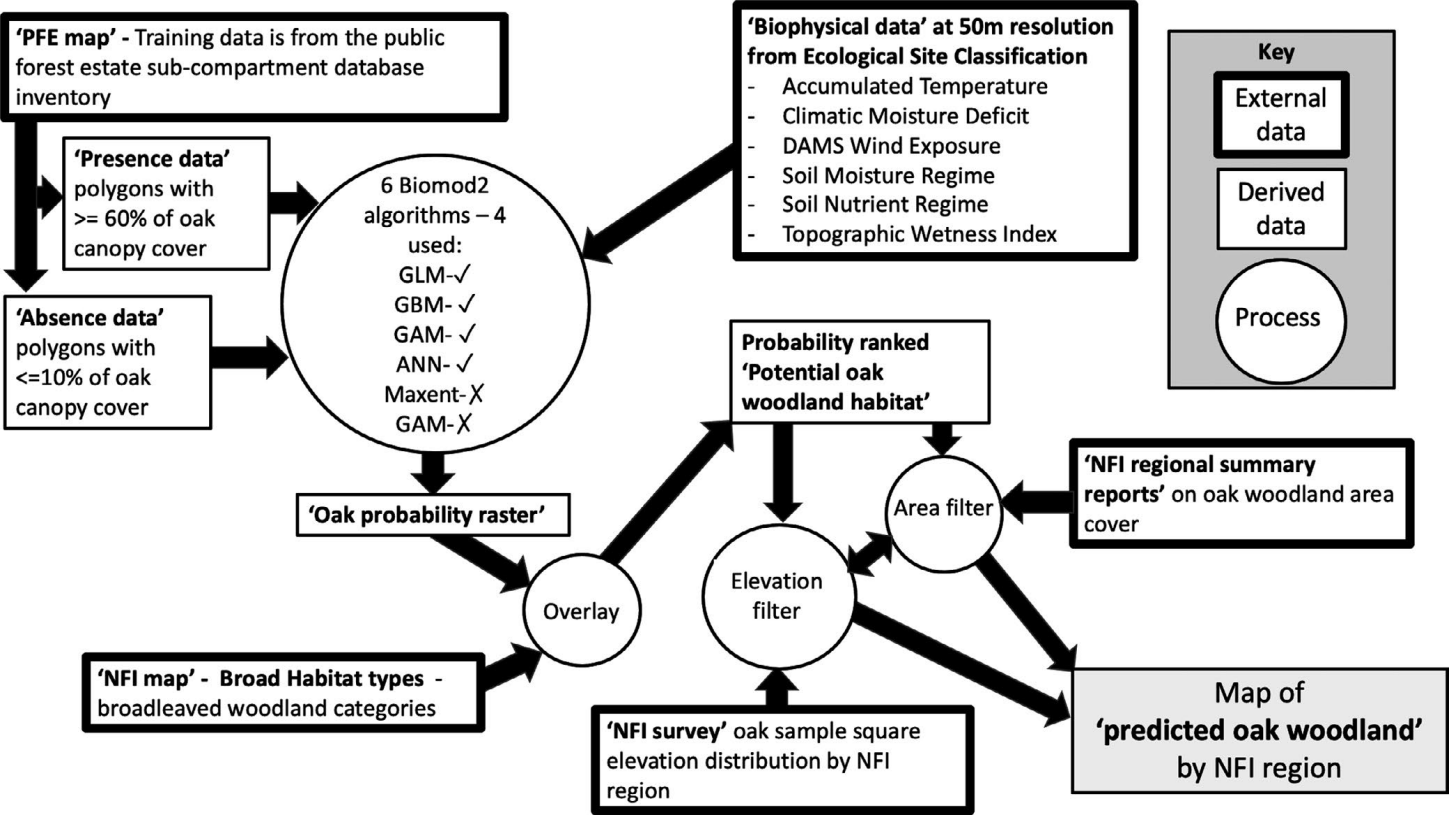
Schematic overview of the study with external data, derived data, and processes shown within the flow of information, to produce a map of “predicted oak woodland”

A flow diagram showing the external data, analysis process, and derived data in the study is provided in Figure 1.

### 2.1 Species presence/absence data

Reliable and standardized data on species occurrence, environmental information, and climatic information are fundamental for modeling purposes. We selected the broadleaved woodland categories from the “NFI map,” which locates the occurrence of all Phase 1 (JNCC, 2016) forest habitat (broadleaved, conifer, mixed woodland, felled, scrub, coppice, restocked broadleaved, restocked conifer), from a 2011 aerial survey. We also used the public forest estate (“PFE map”) subcompartment database polygons for 2011. These data represent forest stands of similar age, species composition, and structure and vary approximately 5–30 ha in spatial extent for England, Scotland, and Wales. Since our modeling approach required a set of oakwood presence and absence records for model training and testing, these were prepared from the 2011 PFE map in the following way.

Oakwood presence in the PFE map polygons was selected containing components of either *Q*. *petraea* or *Q*. *robur* comprising 60% or more of the subcompartment area. Occasionally, the PFE map recorded only “oak,” and this was taken to mean *Q. robur* or *Q. petraea*. Other oak species such as *Q. rubra* were not included in the presence data. Oak absence data were prepared from the PFE map polygons comprised of broadleaved woodland without *Q. robur*, or *Q. petraea*, or “oak,” or where the polygon oak component was a small (10% or less) part of the subcompartment area.

In combination with the PFE map and NFI maps, we used the “NFI survey” of oak woodland sample squares. These data are a representative random‐stratified sample of 0.6% of the British woodland area, consisting of sampled 1 ha woodland squares in 14 NFI regions (Figure S1, supplementary material), including both private and public ownership. For each NFI region, we used the elevation distribution of the NFI sample squares containing oak stands, with a canopy cover greater than 60%. As these data are confidential, they were accessed on our behalf by analysts of the NFI in Britain and made available at the NFI regional level. We used the elevation frequency distribution of all 60% canopy cover oakwood stands occurring in sample squares, to filter our model output results by elevation. Further nonspatial data of the estimated area of stocked native oakwood (*Q*. *robur* and *Q*. *petraea*) from “NFI regional summary reports” (Ditchburn & Ross, 2018) were available for our study. These data include pure, as well as mixed, stands containing oak and provided an estimate of the total area of oak stands by NFI region to additionally filter our model output.

A test validation set of the location of oak woodland sites was created, using the Special Area of Conservation (SAC) oakwood site database (JNCC, 2019), and the ICP Forests Level 1 oak plots (Lorenz & Fischer, 2013), and any of these oak woodlands overlapping any training data were removed. The remaining 64 oak woodlands formed a test validation dataset and covered all NFI regions, although there was a slight bias to western regions of Britain (Figure S1). Thirty‐two percent of sites were in England, 32% in Scotland and 19% in Wales.

### 2.2 Biophysical data

Digital, spatially explicit, biophysical data were prepared from the Ecological Site Classification—ESC (Pyatt et al., 2001), the forest site classification system used in Britain (Ray et al., 2019), summarized in Table S1 (supplementary material). These data were chosen to maintain a consistent link between the study and the forest site classification, which predicts ecological suitability and tree growth of species, including oak woodland under current, and future climates scenarios (Broadmeadow et al., 2005).

The biophysical 50 m × 50 m raster data comprise three climatic variables (calculated for the 30‐year climate change baseline normal period 1961–1990): accumulated “degree‐day” temperature above 5℃ (AT); climatic moisture deficit (CMD); and wind exposure (DAMS—Gardiner & Quine, 2000)—and two soil factors: soil moisture regime (SMR); and soil nutrient regime (SNR)—and the topographic wetness index (TWI), from a 50‐m resolution digital elevation model (EEA, 2017). Spatial raster data for SMR and SNR were derived from a digital map of the major soil sub‐groups, described in soil maps published by the Soil Survey of Scotland, Soil Survey of England & Wales, and the Forestry Commission. The variables AT, CMD, DAMS, and TWI were set as continuous variables, and the factors SMR and SNR were set as integer values, all set to 50 m × 50 m resolution.

### 2.3 Data preparation

The biomod2 package (Thuiller et al., 2016) within the R statistical language (R Development Core Team, 2020) was prepared, and initialized, to randomly sample pixels (50‐m resolution) of the biophysical data associated with broadleaved woodland polygons of the PFE map. The sampling procedure ensured an equal balance of known oak woodland sites (presence data), using half the pixels intersecting oak woodland polygons, and non–oak woodland polygons (absence data) to maintain the prevalence of presence to absence data at 0.5.

Independent predictor variables were tested for collinearity using the R raster package “usdm” (Naimi et al., 2014), to construct a pair‐wise comparison plot showing the degree of correlation between biophysical variables. The R “car” package (Fox & Weisberg, 2019) was used in a regression analysis, to check which correlated variables contributed most of the explained variance. The best predictors from the collinearity test were used in the analysis.

### 2.4 Biomod2—Species distribution modeling

Biomod2 provides an ensemble platform of ten SDM algorithms, and we initially used just six of these as ensemble candidates. These were generalized linear model (GLM), gradient boosted machine learning (GBM), generalized additive model (GAM), artificial neural networks (ANN), random forest classifier (RF), and maximum entropy model (MAXENT). Algorithms that were not able to fit all the NFI regions successfully were removed, leaving just four after removing GAM and MAXENT. This provided the same four algorithms to model all the NFI regions.

Single‐algorithm, oak probability raster results were averaged for an ensemble prediction. The literature suggests absences may be sampled by random selection to combine with presence records (Barbet‐Massin et al., 2012). Given the large difference between the total number of presence records available and the potential candidate absence records, it was necessary to balance the presence and absence data, and at the same time consider all the environmental variation of an NFI region. Therefore, we randomly sampled 15 replicates of absence data, with the same number of points as the presence data. The use of more than one absence dataset allowed us to consider a larger ecological environment than a single absence dataset. The 15 sets of absence data were created using a random subset of the total absence pixels from broadleaved woodland patches (NFI map) that coincided with the PFE map polygons of forest without the presence of oak. To avoid overfitting, a cross‐validation procedure was applied using 50% data partition (Lobo & Tognelli, 2011). In biomod2, the cross‐validation procedure was repeated 30 times for each of the 15 presence–absence groups.

Using this procedure, we calculated the true skill statistic (TSS) for each replicate, run, and algorithm. Evaluation was made on the size of the true skill statistic (TSS). The TSS is reported as a value between 0 and 1, with 1 indicating excellent prediction (Allouche et al., 2006). A single oak probability raster prediction was calculated as the weighted mean of the four algorithm predictions using TSS scores as a weighting factor. The SDMs were parameterized separately for each of the 14 NFI regions in Britain; this allowed separate parameterization of models for regional variations in management, site selection, and site condition, for oak woodland stands in each NFI region.

### 2.5 Additional data and methods to predict oak spatial distribution

The probability raster of oak suitability for Britain shows land that has biophysical properties suited to oak woodland. We refined our prediction of the oak spatial distribution by masking and filtering (two steps) the probability raster with NFI data.

#### 2.5.1 Masking

Separately for each NFI region, the output oak probability raster was masked by clipping to the broadleaved woodland category polygons of the NFI map (broadleaved woodland/mixed predominantly broadleaved woodland/coppice/coppice with standards) and the value of all the 50‐m pixels not occurring within the broadleaved woodland polygons were set to zero. We ranked the pixels from the highest to the lowest probability value separately for the 14 NFI regions.

#### 2.5.2 Filtering

In each region, the total area covered by oak stands had been separately derived by the NFI (Ditchburn & Ross, 2018) and for this work the elevation distribution of oak woodland was extracted from the NFI survey sample squares. Two maps of the spatial distribution of oak woodlands were derived using steps 1 to 2. Further, we tested whether a manual adjustment of the SDMs was necessary (step 3). Finally, a comparison of products was performed using steps 4 and 5. The 5 filtering steps were as follows:

1. The area of ranked pixels was accumulated up to the total area of stocked oak woodland published for each region (Ditchburn & Ross, 2018).
2. We modified the method with an additional step, to match the elevation distribution of the oak NFI survey sample squares, using elevation classes of 10 m (i.e., 0–9, 10–19, 20–29). This analysis took the highest probability ranked pixels in an elevation class from any polygon until the number of pixels in the elevation class matched the elevation class of the oak NFI survey sample squares. This was repeated for each elevation class in turn to produce a second spatial map of likely oak‐dominated woodlands of 60% canopy cover (or greater).
3. We tested whether a manual adjustment of the SDMs was necessary. For this, the oak training dataset used in the biomod2 analysis was included prior to the pixel selection in step 2. These data were known oak stands from the PFE map of the public forest estate. A probability score of 1 was manually allocated to all pixels covered by this stratum to ensure they would be selected as oak woodland pixels. The average difference between the manually adjusted layer and the biomod2 output, prior to manual adjustment, was calculated.
4. We analyzed the similarity of the oak probability raster distributions of pixels (using a nonparametric Kolmogorov–Smirnov test) within NFI map broadleaved woodland polygons separately with the pixels constrained only by total area of oak (step 1) and pixels constrained by total area of oak and by elevation distribution (step 2).
5. We tested the coincidence of predicted oak woodland polygons with an independent set of known oak woodland locations (Figure S1). The percentage of correct predictions to incorrect predictions provided by the area and the area plus elevation filtered models were compared using an exact binomial test in the R package “binom” (R Development Core Team, 2020).

## 3 RESULTS

### 3.1 Model structure

A list of the variables used to train the fourteen NFI regional models is given in Table 1. Table 1 indicates the variables removed, based on a collinearity test, and the number of pixels sampled as presence data. It also provides an indication of the important environmental variables associated with the presence of oak woodland in each NFI region. Topographic wind exposure (DAMS), soil moisture regime (SMR), soil nutrient regime (SNR), topographic wetness index (TWI), accumulated temperature (AT), and climatic moisture deficit (CMD) were all selected for inclusion in one or more NFI regions. However, it should be remembered that DAMS is directly influenced by elevation (DEM), and that AT and CMD are inversely related to elevation. The number of presence pixels varied among NFI regions according to the spatial distribution of the target species.

**TABLE 1:** The independent spatial data used in the fourteen national forest inventory regions, the data removed resulting from collinearity test, and the number of oak wood presence data sampled in each region; each of the 15 absence datasets in an NFI region exactly matched the number of presence pixels

Independent variables used in the model						Variables removed from model following collinearity test			No. presence pixels selected
NFI region	Variable list					Variable list			
1	DEM	DAMS	TWI	SNR	SMR	AT	CMD	–	16,317
2	DEM	DAMS	TWI	SNR	SMR	AT	CMD	–	18,113
3	AT	DAMS	TWI	SNR	SMR	CMD	DEM	–	2,402
4	CMD	TWI	SNR	SMR	–	AT	DAMS	DEM	798
5	DAMS	TWI	SNR	SMR	–	CMD	AT	DEM	429
6	DAMS	CMD	TWI	SNR	SMR	AT	DEM	–	1847
7	DAMS	AT	TWI	SNR	SMR	CMD	DEM	–	4,773
8	DAMS	AT	TWI	SNR	SMR	CMD	DEM	–	13,390
9	DAMS	AT	TWI	SNR	SMR	CMD	DEM	–	4,033
10	DAMS	AT	TWI	SNR	SMR	CMD	DEM	–	4,096
11	DAMS	DEM	TWI	SNR	SMR	AT	CMD	–	4,746
12	DAMS	AT	TWI	SNR	SMR	CMD	DEM	–	25,944
13	DAMS	CMD	TWI	SNR	SMR	AT	DEM	–	1,065
14	DAMS	AT	TWI	SNR	SMR	CMD	DEM	–	36,585

Abbreviations: AT, accumulated temperature; CMD, climatic moisture deficit; DAMS, digital wind exposure; DEM, digital elevation model; SMR, soil moisture regime; SNR, soil nutrient regime; TWI, topographic wetness index.

### 3.2 Evaluating performance of biomod2 algorithms

**FIGURE 2:**
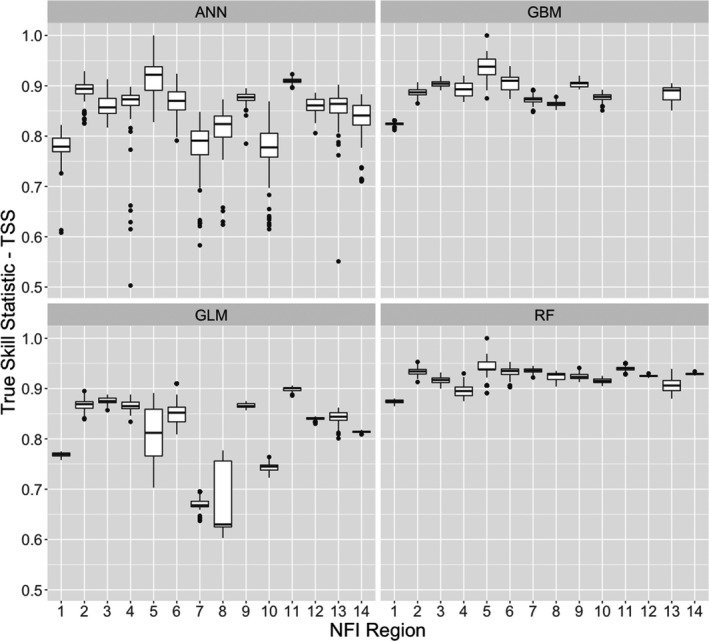
Comparison of TSS scores showing model fitting performance for the biomod2 algorithms: artificial neural networks (ANN), generalized linear model (GLM), gradient boosted machine classifier (GBM), and random forest classifier (RF), for each of the 14 NFI regions for which the four models were separately parameterized. NFI regions are as follows: 1‐North West England, 2‐North East England, 3‐Yorkshire and Humber, 4‐East Midlands, 5‐East England, 6‐South East England, 7‐South West England, 8‐West Midlands, 9‐North Scotland, 10‐North East Scotland, 11‐East Scotland, 12‐South Scotland, 13‐West Scotland, and 14‐Wales

We generated TSS values based on cross‐validation of the model fitting procedure calculated separately for each NFI region. This showed that classification methods GBM and RF performed better than the statistical GLM method or the ANN method (Figure 2). The other two algorithms (MAXENT and GAM) either failed to converge or showed poor stability and were removed from the analysis.

The RF method produced high and stable TSS values (stability shown by a smaller variation about the median TSS) with a median value of 0.87 (or higher), in each of the replicate runs for an NFI region. The RF median TSS ranged between 0.87 for NFI region 1 and 0.93 for region 5. The GLM method produced lower median TSS values compared with the RF, GBM, and ANN methods, with less consistency. GBM performance was very good, although there were three NFI regions where the model failed to converge: East Scotland (Region 11), South Scotland (12), and Wales (14). The ANN method provided very high TSS values for six of the NFI regions, including regions 11 and 12, where the GBM failed to converge, but ANN TSS results were less stable in regions 1, 7, and 10. The ANN method produced high TSS values in North East England (Region 2), East Midlands (4), East England (5), South East England (6), North Scotland (9), and East Scotland (11). This is despite a very small area of oak woodland predicted in North East England, North Scotland, and East Scotland (Regions 2, 9 & 11). The GLM method produced slightly lower median TSS values than GBM and RF. Apart from the NFI regions East England (Region 5) and West Midlands (8), the GLM was more stable than the ANN algorithm. For each of the NFI regions, the ensemble TSS‐weighted mean probability of oak occurrence was comprised of at least two high stable values and two less stable, lower, or no data values.

### 3.3 Using biomod2 results with additional data to predict oak spatial distribution

**FIGURE 3:**
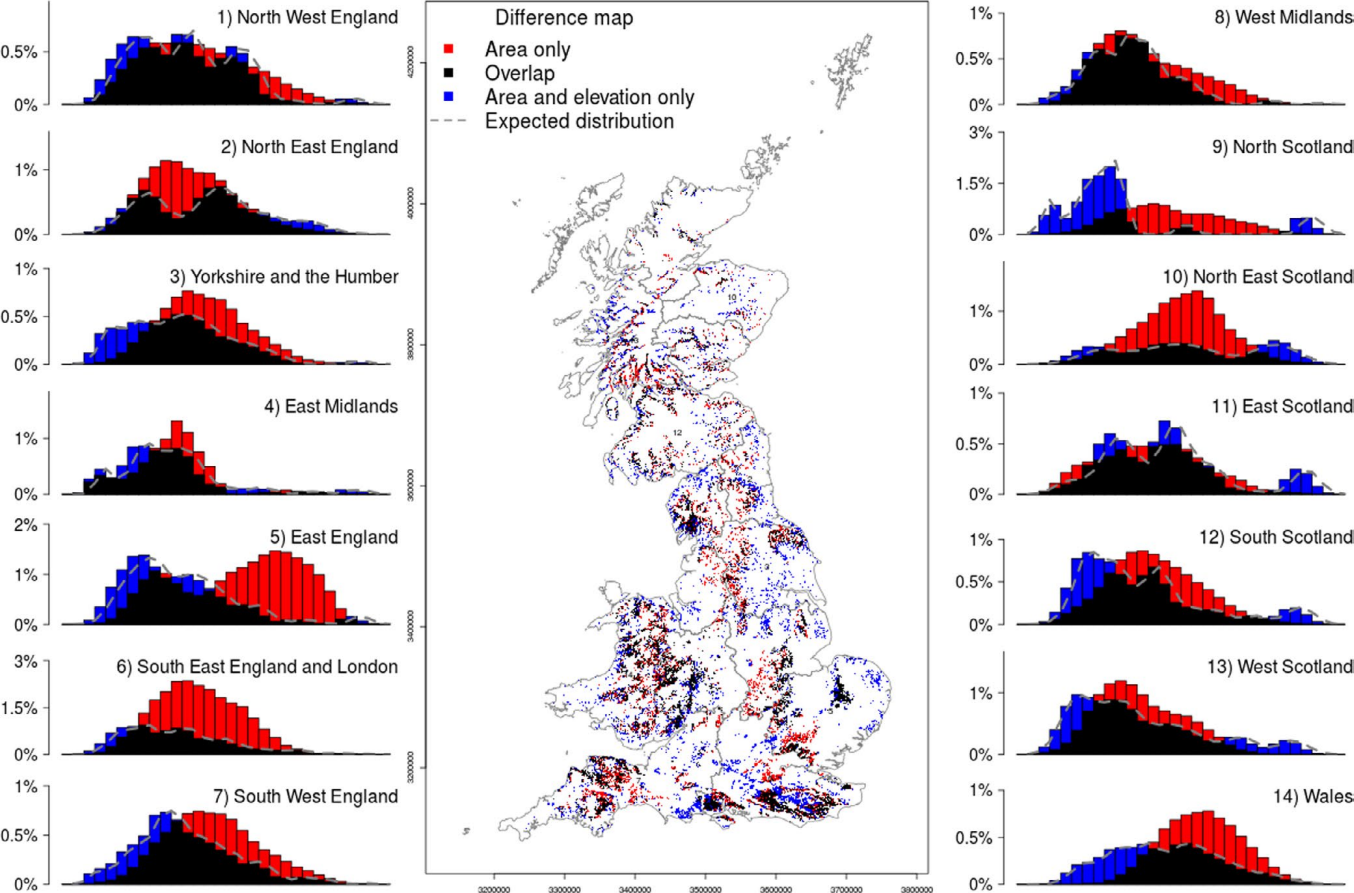
Spatial distribution models obtained for Britain by filtering the biomod2‐predicted results using: the published National Forest Inventory (NFI) area only (red histograms and map points) and the NFI area plus the elevation distribution based on the NFI survey sample square data (blue histograms and map points), area in black shows the coincidence of points in each filtering method. Each bar of the histogram represents a 10 m elevation class

The results of the novel filtering procedure applied to the biomod2 probabilistic map are presented (Figure 3) as histograms and spatial distributions of predicted *Q*. *petraea* and *Q. robur* woodlands in Britain. The maps show and compare the distribution of oak from probability ranked biomod2 pixels constrained by the NFI regional area of oak (Figure 3—red points) and the probability ranked pixels constrained by area and the elevation distribution (Figure 3—blue points), with overlapping areas of each filtering method (Figure 3—black points). The central map portion of Figure 3 is reproduced as Figure S2 (supplementary material). Fourteen histograms, showing the frequency of pixels in 10 m elevation categories, surround the map (using the same color key) and show the elevation distributions for the two filtering methods. The histograms show the combined area and elevation method was effective in every NFI region.

Several NFI regions showed the elevation distribution of the biomod2‐projected area off‐set or skewed from that reported in the NFI survey sample square data. This was particularly noticeable in North East England (2) and Yorkshire and Humber (3), and was less prominent in other NFI regions. The NFI regions with an elevation distribution of biomod2 pixels similar to the sample squares were East Midlands (4), West Midlands (8), East Scotland (11), South Scotland (12), and West Scotland (13). These three regions in Scotland had very small, predicted areas of oak (Ditchburn & Ross, 2018), whereas in England, the regions with low stocked areas of oak (North East England and Yorkshire and Humber) showed the least similar elevation distribution of pixels with sample square data.

**FIGURE 4:**
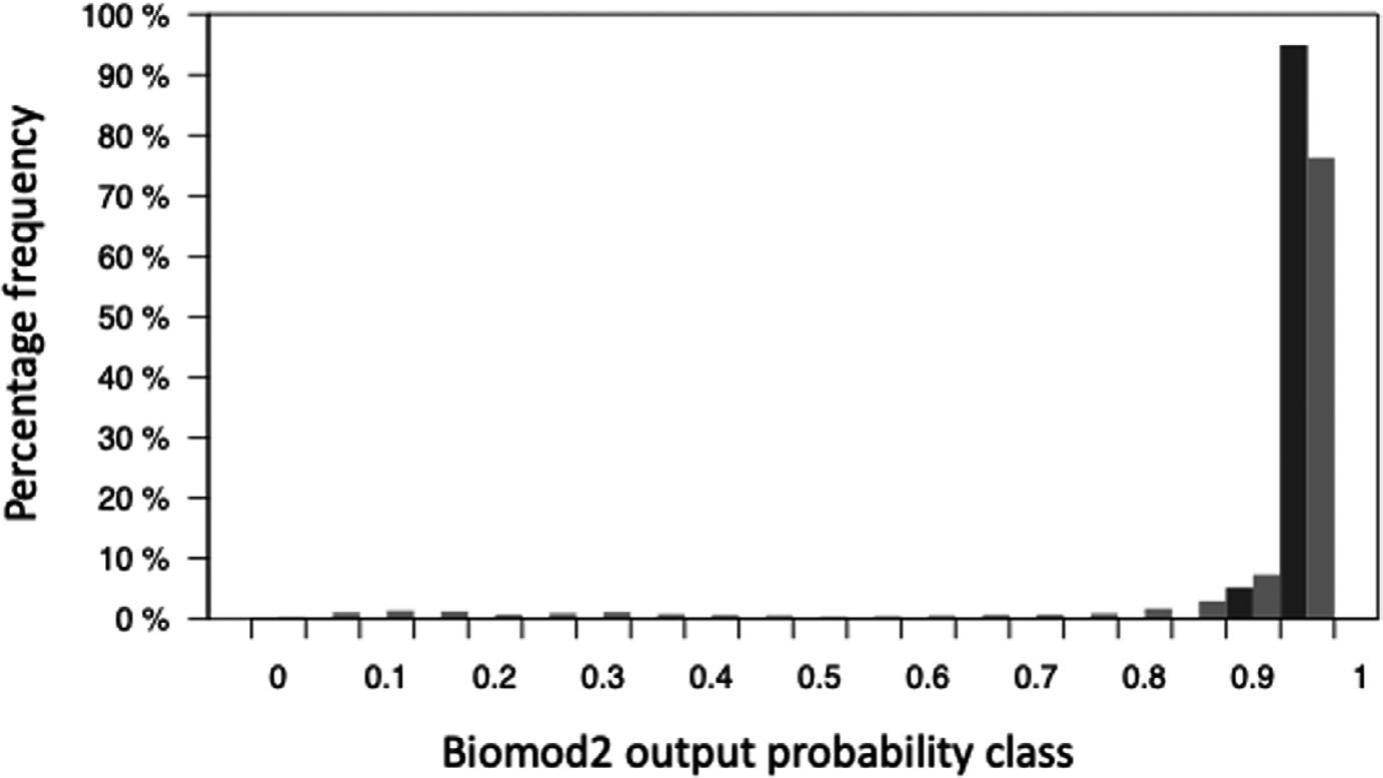
Comparing the biomod2 TSS‐weighted mean output oak probability raster distribution of pixels for NFI maps of Indicative Forest Type (IFT) broadleaved woodland polygons in Britain, filtered in two ways: (i) NFI oak area (black bars) and (ii) NFI oak area and NFI survey sample square elevation distribution (gray bars)

A Kolmogorov–Smirnov test showed a highly significant difference (*p*‐value ≤ .001, *D*‐value 0.28) between the biomod2 oak probability rasters “filtered by area” and “filtered by area and elevation.” Figure 4 shows that forcing pixels into the elevation distribution of the NFI survey sample squares caused a low probability tail in the distribution compared with the area‐only filter. These data show the main difference between the highest ranked oak woodland probability raster values in broadleaved woodland (NFI map) polygons from the biomod2 results, and the same results adjusted by additional NFI survey sample square information.

The lowest value of a pixel passing the “area only” filter had a value of 0.92 in East England, whereas the lowest value of a pixel passing the area plus elevation filters was 0.03 in NFI region Yorkshire and Humber.

Our assessment to see whether the resetting of the oak probability raster value of biomod2 pixels to one (certain probability), for the public forest estate (PFE map) oak training data, showed only a very small effect on the pixels selected as predicted oak woodland, compared with not changing the probability raster of pixels on known oak sites. The average difference between the adjusted value (*p* = 1) and the biomod2 output probability value was .0012. Consequently, the pixels we adjusted to one were already among those with the highest probabilities in all the NFI regions. This result showed the input data were good enough to characterize the ecological niche of the target species in all NFI regions.

Our independent test comparing predicted oak woodlands with independent oak woodland locations not used in the training data gave a good result. The performance of the area filtering method revealed a 55% coincidence success rate with a confidence interval between 42% and 67%. The area filter correctly predicted 35 out of 64 independent woodlands with a *p*‐value of 0.5323. In contrast, the performance of the area plus elevation filter method predicted 83% coincidence success rate with a lower and upper confidence interval of 71% and 91%, respectively. The double filter correctly predicted 53 of 64 independent woodlands, with a *p*‐value <.0001. The “coincidence success” is the “true presence rate” or “sensitivity” of the model as commonly used in the literature. The area plus elevation filter showed a significant improvement in correctly allocating “predicted oak woodland” to the broadleaved woodland NFI map polygons.

## 4 DISCUSSION

In our oak woodland case study, SDMs using a biomod2 approach provide a robust and realistic oak probability raster of managed secondary and ancient semi‐natural woodland types. Several algorithms in the biomod2 ensemble performed well with our data, showing four methods that converged successfully, giving stable TSS results. In recognition that in Britain, for secondary and ancient semi‐natural woodlands in heavily managed landscapes, the most suitable sites may not always be occupied by the most suitable woodland type (or any woodland), we developed a method to adjust the SDM approach to help correct this problem. Using additional information sources, such as the NFI maps and elevation of NFI survey sample square data, we were able to mask and filter the most likely oak probability raster pixel values to form a map of the extent and location of oak woodland type which reflects the current NFI survey sample squares woodland type distribution. An independent set of oak woodland locations not used for training the model showed reliable model predictions.

Previous work (Hill et al., 2017) reported that an SDM approach for locating oak trees and their abundance in the landscape can provide very valuable data for ecologists. Our objectives were rather different from the Hill et al. (2017) study, in that we were interested in identifying predominantly oak woodlands with 60% canopy cover at high resolution, including plantation origin and heavily managed ancient oak woodlands. They produced maps of 1‐km resolution for the abundance of oak (ha.km^−2^) that cover the same type of oak woodland stands, and in addition, they included oak tree abundance outside NFI‐mapped woodland stands. Our objective was to precisely predict which of the indicative forest type NFI map polygons were most likely to contain oak stands. We wished to develop an approach that when combined with climate projections, would help recognize where, and how, forest management adaptation might be needed to reduce the impacts of abiotic and biotic threats to oak woodlands under climate change. Our method of employing an SDM (biomod2) approach has some similarities with the study of Hill et al. (2017), but our ensemble data sources differ.

Our use of an ensemble of four algorithms within biomod2 provided complete coverage of oak woodland predictions with very high TSS predictive performance, from all the independently parameterized NFI regions in our study. This is an important result from an ensemble, or concensus method, to reduce uncertainty (Zhu & Peterson, 2017) associated with the situation dependence of environmental data on the predictive performance of individual algorithms (Hao et al., 2020; Shabani et al., 2016). By fitting algorithms separately to each NFI region, model parameterization adjusted the situation dependence to regional environmental variables (Grenouillet et al., 2011).

The risk of failing to generate a prediction from a single model was reduced by the ensemble approach. None of the tested algorithms was the best across all the NFI regions. Studies have shown that an ensemble technique provides advantages over a single algorithm approach (Araújo & New, 2007; Grenouillet et al., 2011; Shabani et al., 2016) and that the TSS is a more appropriate measure of accuracy, in which oak probability pixel values are not ignored, and commission and omission errors are equally weighted (Lobo et al., 2008). We combined data partitioning (50% training ‐ 50% validation) producing 30 random selections from the presence data with each of the 15 balanced absence datasets. Thus, individual model runs used fewer presence and absence points to avoid overfitting, and the combined 450 model evaluations were used to calculate a TSS‐weighted mean oak probability raster score based on a large absence dataset.

The area plus elevation filter showed a significant improvement in the correct allocation of oak woodlands, as shown using an independent dataset of private oak woodland locations. Our method has shown the occurrence of pixels with a very high probability for oak suitability that are unoccupied by woodland or occupied by another nonbroadleaved woodland type. This raises the possibility of using the unfiltered output raster for predicting where oak woodland restoration or reforestation could be ecologically suitable.

In Britain, most types of oak woodland are suited to sites with medium to rich fertility (Pyatt et al., 2001), although some oak woodlands do occur on poorer sandy textured soils. The oak woodland types are well described in Britain's National Vegetation Classification (Rodwell, 1991). The more common *Quercus robur* is suited to heavier textured, clay soils in the lowlands of England and Scotland, whereas *Quercus petraea* is more commonly found in the west of Britain on freely draining soils, but with higher precipitation. The primary reason for parameterizing separate biomod2 models for each NFI region was to account for these regional differences in the site types of oak woodland communities. Indeed, the bimodal histograms seen in some of the NFI regions are very likely to represent different types of oak woodland in a region. Both upland and lowland oak woodlands have been characterized by the woodland NVC.

Oak decline has affected oak stands across Europe (Keča et al., 2016; Landmann, 1993; Oosterbaan & Nabuurs, 1991; Rozas & García‐González, 2012; Thomas et al., 2002) and has also been described in Britain (Brown et al., 2018; Denman et al., 2014; Gibbs & Greig, 1997; Osmaston, 1927). This study will provide information about the effects of climate change on oak woodland sites, particularly the effects due to extreme events that may predispose oak to decline.

While the location of private woodlands recorded in the NFI survey remains confidential, this study shows how to produce a reasonably realistic oak woodland distribution map for a country, without additional expensive field survey or remote sensing analysis. Our approach represents a valuable case study that will be applied to other managed woodland types where the exact location of stands is unknown, but site data on a sample of stands is available. This situation is common in Europe in countries where the National Forest Inventory data quality is limited, and models (e.g., EFIScen) give results at a low resolution (Nabuurs et al., 2018; Verkerk et al., 2019). Vidal et al. (2016) report that at a European level a greater research effort of the “nature, extent, and effects of climatic change” on forest ecosystem services is needed. At a fine resolution, our method allows an increased understanding of site‐type interactions with future climate projections and impacts on ecosystem services (Ray et al., 2015, 2019), and this may inform forest policy strategies to help target climate change adaptation management in forestry practice.

## 5 CONCLUSIONS

We found that SDM ensemble algorithms in the “biomod2” R package were able to accurately predict the location of oak woodland types. This applied to oak stands in long‐established native woodlands (ancient semi‐natural woodlands) and more recent secondary woodlands. The ensemble approach smoothed variation in model performance resulting from regional interactions between predictor variables and forest stands.

In assessing the different approaches of comparing an area filter and an area plus elevation filter, we found that the combination of an area plus elevation filter significantly improved the prediction success for oak woodland in Britain. The elevation distribution was from the NFI survey square data for oak woodlands in each NFI region, and it provided an important filter for the ranked probabilistic pixels of oak woodland from biomod2. The unmasked SDM oak probability raster of pixels indicated potential sites suitable for oak woodland expansion (land‐use change) or the selection of oak as a potential replacement species.

We anticipate that biomod2 will help improve our knowledge of the distribution of other woodland types, by repeating this study for different tree species. This will form an important initial step to tailor recommendations and guidance on forest resilience planning and management for specific tree species–site‐type interactions under climate projection scenarios. Biotic and abiotic stress on *Q*. *petraea* and *Q*. *robur* increases under climate change, and it is important to evidence the predisposing environmental drivers and provide spatially explicit predictions of vulnerability to plan adaptation management responses.

Accurately mapping tree species distributions is a key step in meeting these ambitions. Our work has offered a method to do this in a relatively inexpensive and timely way to deliver the information quickly. The approach could be applied to other broadleaved woodland types and to other forms of habitat to help inform climate change adaptation policy and practice (Bosso et al., 2017; Thorne et al., 2017).

## CONFLICT OF INTEREST

None declared.

## AUTHOR CONTRIBUTION

**Duncan Ray:** Conceptualization (equal); Investigation (equal); Supervision (equal); Writing‐original draft (equal). **Maurizio Marchi:** Formal analysis (equal); Methodology (equal); Software (equal); Validation (equal); Writing‐review & editing (equal). **Andrew Rattey:** Formal analysis (equal); Software (equal); Validation (equal); Visualization (equal). **Alice Broome:** Methodology (equal); Resources (equal); Writing‐review & editing (equal).

## Supporting information

Appendix S1

## Data Availability

Data used in the study are publicly available and accessible and held on the UK government website: https://www.gov.uk/guidance/access‐forestry‐commission‐datasets#spatial‐datasets‐for‐use‐in‐a‐geographical‐information‐system‐gis. Biophysical variables for the Ecological Site Classification are publicly available and accessible and can be obtained from http://www.forestdss.org.uk/geoforestdss/. The test validation oak woodland sites designated as special areas of conservation are available from the UK Government Agency Joint Nature Conservation Committee at https://jncc.gov.uk/search?q=Natura+2000
