# Supplementary material for: A multi‐data ensemble approach for predicting woodland type distribution: Oak woodland in Britain
Source: Ecol Evol. 2021 Jun 21;11(14):9423–34. doi: 10.1002/ece3.7752 (PMC8293729; doi:10.1002/ece3.7752)
Supplement: Supplementary file 1 — Appendix S1 [file ECE3-11-9423-s001.docx]

Supplementary Material to “Spatial prediction of oak woodland”

Figure S1 Location of the National Forest Inventory (NFI) Regions in Britain, and the location independent oak woodland sites used for testing the results of the study; oak woodlands that were not included in the training data set. NFI regions are: 1) North West England, 2) North East England, 3) Yorkshire and Humber, 4) East Midlands, 5) East England, 6) South East England and London, 7) South West England, 8) West Midlands, 9) North Scotland, 10) North East Scotland, 11) East Scotland, 12) South Scotland, 13) West Scotland, 14) Wales


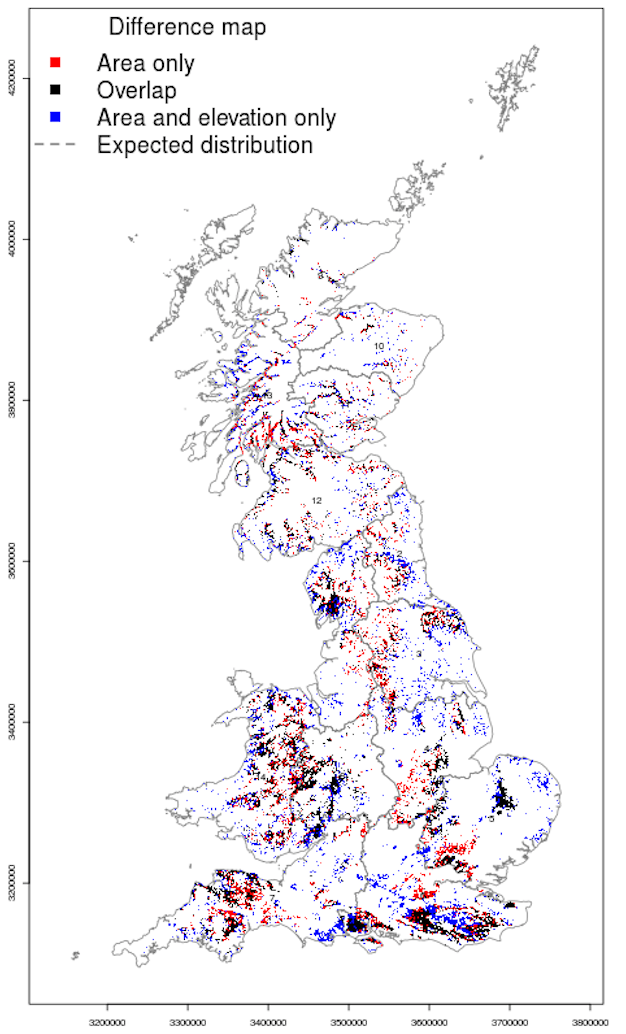


Figure S2 Spatial distribution model results of oak dominant woodlands (>=60% canopy cover) obtained for Britain by filtering biomod2 predicted results using: the published National Forest Inventory (NFI) area only (red map points) and the NFI area plus the elevation distribution based on the NFI survey sample square data (blue map points), and areas in black showing the coincidence of points in each filtering method

Table S1 Description of the spatial data used from Ecological Site Classification, the forest site classification system used in Britain. All data are spatial at a resolution of 50x50m
